# A global call for family-centered ICU care

**DOI:** 10.1016/j.lana.2025.101036

**Published:** 2025-02-21

**Authors:** Bradley A. Firchow

**Affiliations:** Rural Physician Leadership Program, University of Kentucky College of Medicine, Morehead, KY, USA

Brandao Barreto, Luz, and Gusmao-Flores compellingly highlight the exclusion of children from intensive care units (ICUs) and the need for a more inclusive, family-centered approach.^1^ The authors’ position is affirmed, along with their comprehensive examination of the emotional impact on children and the call for greater preparation and support from healthcare teams. This discourse can be further enriched by broadening the context through a global lens, highlighting how inclusive ICU policies promote emotional well-being and foster more cohesive family units across healthcare settings.

Globally, the adoption of family-centred ICU care remains highly inconsistent, with cultural norms, infrastructure challenges, and outdated policies acting as significant barriers.^2^ While Brazil lags with only 12.5% of ICUs permitting unrestricted child visitation, countries such as France have made considerable progress, with over 59% adopting open visitation policies.^3^ However, even in nations with progressive policies, implementation often varies widely depending on regional resources and institutional culture. In the United States, for instance, ICU visitation guidelines are primarily left to individual institutions, resulting in significant variability and missed opportunities for more inclusive care practices.^4^

Research highlights that children benefit emotionally and psychologically from visiting critically ill family members in the ICU, with reported outcomes including increased feelings of involvement and reduced emotional distress. Structured visitation experiences can also help children process their experiences, fostering a sense of security and connection with their loved ones.^5^

It is crucial to address concerns raised by healthcare professionals—such as infection control, emotional distress, and operational challenges—through education, policy reform, and the development of practical resources. Training interdisciplinary teams to engage with children and families in a meaningful way, as suggested by the authors, is a necessary step forward. Innovative approaches such as the use of visual aids, child-friendly ICU models, and dedicated family liaisons can bridge gaps in communication and ensure a more inclusive, humane experience for all family members.^2^

In a world where family-centred care is recognized as essential, excluding children from the ICU undermines both patient and family well-being. It is time to move beyond restrictive policies and embrace comprehensive strategies that truly reflect the needs of all family members—children included.

## Declaration of interests

The author declared no conflicts of interest.
